# Publication Trends of Research on Gallbladder Cancer During 2001–2021: A 20-Year Bibliometric Analysis

**DOI:** 10.3389/fonc.2022.932797

**Published:** 2022-07-11

**Authors:** Wentao Sun, Wenze Wan, Zhihui Gao, Tao Suo, Sheng Shen, Houbao Liu

**Affiliations:** ^1^ Department of General Surgery, Zhongshan Hospital, Fudan University, Shanghai, China; ^2^ Biliary Tract Disease Institute, Fudan University, Shanghai, China; ^3^ Shanghai Engineering Research Center of Biliary Tract Minimal Invasive Surgery and Materials, Shanghai, China; ^4^ Department of Nuclear Medicine, Zhongshan Hospital, Fudan University, Shanghai, China

**Keywords:** Gallbladder cancer (GBC), bibliometric analysis, publication, citation, research

## Abstract

Gallbladder cancer (GBC) is one of the lethal cancers with an extremely poor prognosis. In the recent 20 years, research on GBC has developed rapidly. Here we aim to perform a systematical bibliometric analysis on the current foci and status of GBC research. This study analyzes trends in GBC research and compares contributions from different countries and regions, institutions, and authors. All publications in GBC research from 2001 to 2021 in the Web of Science Core Collection (WoSCC) database were collected. Microsoft Excel 2010 and GraphPad Prism 9 were used to analyze publication data and publication trends. VOSviewer 1.6.17 was adapted to generate a visual network of keywords in surgical training research. A total of 3,323 publications were included. China was the most productive country, with the highest number of publications (n = 900, 27.08%). Shanghai Jiaotong University and Roa JC were the most productive institution and authors, contributing 215 and 89 publications, respectively. Keywords were classified into five clusters, each representing a key topic. The main clusters of GBC are related to surgery therapy, mechanism research-related study, and non-surgery therapy, while migration is the current hotspot of GBC research. The scientific progression of GBC research over the past two decades was comprehensively analyzed by this bibliometric study. Finding deeper mechanisms in the migration of GBC cells, new biomarkers, and highly effective nomograms will be the major problems and directions in the future.

## Introduction

Gallbladder cancer (GBC) is the most common cancer in biliary tract malignancy, accounting for 80%–95% of biliary tract malignancy by autopsy studies (1). It is one of the most malignant cancers due to its latency, late clinical detection, and very dismal prognosis (2). The incidence of GBC varied among regions. East Asia, East Europe, and Latin America contribute the highest incidence of GBC (3), which may result from environmental exposures, genetic predisposition, and regional intrinsic risk factors (1). Radical surgery remains the only potentially curative treatment, but only 10% of patients have the opportunity to have radical resection (2). Most patients need therapy including chemotherapy based on gemcitabine as a first-line regimen and radiotherapy (4, 5). However, the median overall survival is less than 1 year and the 5-year survival rate remains low at approximately 5% (6–8).

Due to the poor prognosis, GBC has been the subject of extensive research over the past two decades. Emerging therapy regimens like targeted therapy and immunotherapy show potential efficacy. For example, regorafenib, a multitargeted receptor tyrosine kinase inhibitor, significantly improved PFS in patients with previously treated metastatic/unresectable biliary tract cancer (BTC) including GBC, but the effect on GBC needs more subgroup analysis (9). Patients with refractory BTC or MSI-H/dMMR tumors may benefit from immune checkpoint inhibitors like pembrolizumab and nivolumab (10, 11). Moreover, better understanding of the mechanism in GBC has emerged over the years. Whole-exome sequencing, targeted gene sequencing, and single-cell RNA sequencing depict an atlas of GBC at the molecular level and uncover the essential factors and molecular mechanism in gallbladder carcinogenesis (12–14).

Bibliometric analysis has become a popular and reliable method to evaluate detailed trends in a certain field over time. It applies literature metrology characteristics to measure contributions in a certain field and predict the trends and developments. Till now, there is no bibliometric analysis that focused on GBC. To better retrospect the development and contributions and prospect the future hot spots in GBC research, we present a comprehensive and in-depth analysis on the current state of GBC research based on Web of Science Core Collection (WoSCC) data and provide an insight into the direction and trend in GBC research.

## Materials and Methods

### Data Collection and Search Strategies

We used the WoSCC database to collect literature data. All searches were completed on March 20, 2022. The filtering strategy was set to “(TI=(gallbladder tumor) OR TI=(gallbladder cancer)) OR TI=(gallbladder carcinoma) OR TI=(gallbladder malignancy).” The inclusion criteria were as follows: 1) language: English; 2) publication years: 2001–2021. A total of 3,756 publications were included, while the exclusion criteria were that the types of documents were letters, editorial materials, corrections, books, data sets, proceedings papers, retractions, and retracted publications. A total of 3,323 publications were finally retrieved. Full records and cited references of all the publications that met the inclusion and exclusion criteria were exported and downloaded as plain text files.

### Bibliometric Analysis

All of the publications included in the study were described and analyzed using WoSCC. Denoting that a scholar or region has published H papers that have each been cited at least H times, the H-index has been widely accepted as a measure of scholar’s or region’s scientific research influence. Thus, citations and the H-index which were obtained from the WoSCC database could comprehensively reflect the impact of a scholar or region. The top journals, institutions, and authors and their number of publications were also retrieved from the WoSCC database.

### Data Analysis

Relative research interest (RRI) was calculated as the number of publications in a certain field divided by all publications regardless of field. Using Microsoft Excel 2019, the prediction model was generated to predict future publication trends based on the formula f(x)=ax3+bx2+cx+d. GraphPad Prism 9 was utilized to analyze the quantity and quality of publications in countries, institutions, and journals. VOSviewer was used to visualize a bibliometric network among the keywords from titles and abstracts. All highly reoccurred keywords were grouped into clusters distinguished by different colors.

## Results

### Countries’ Contributions to Global Publications

**Figure 1:**
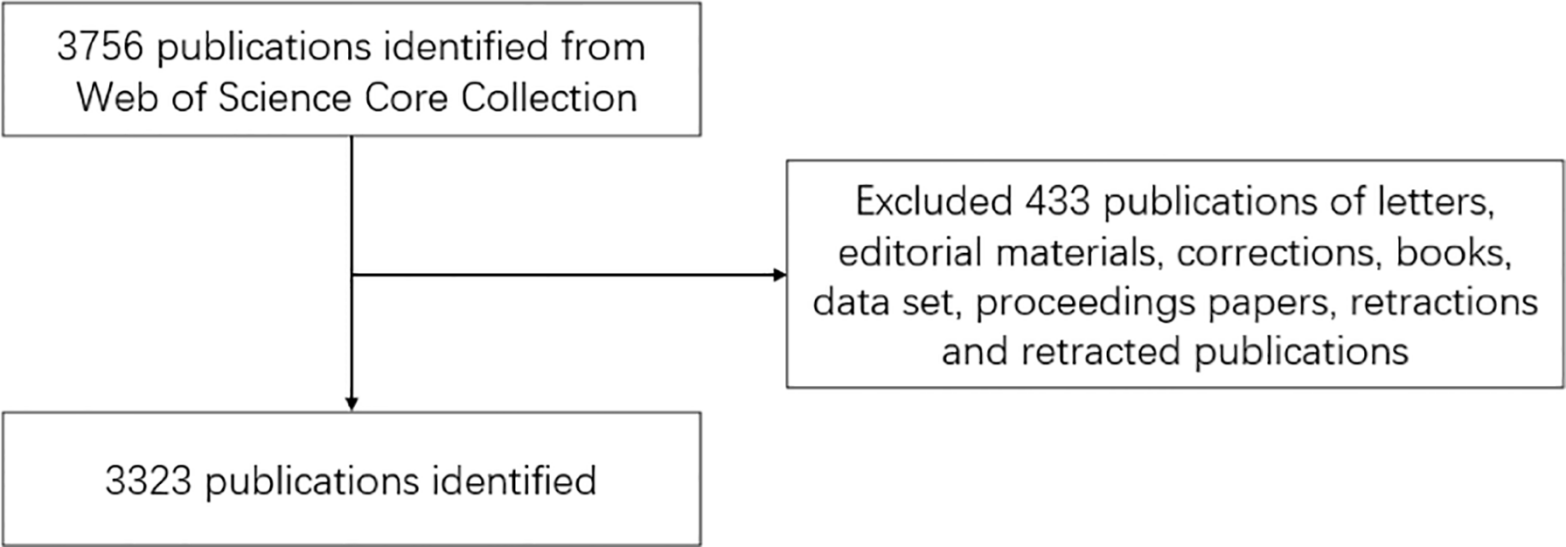
Flow diagram of the gallbladder cancer research inclusion and exclusion process.

**Figure 2:**
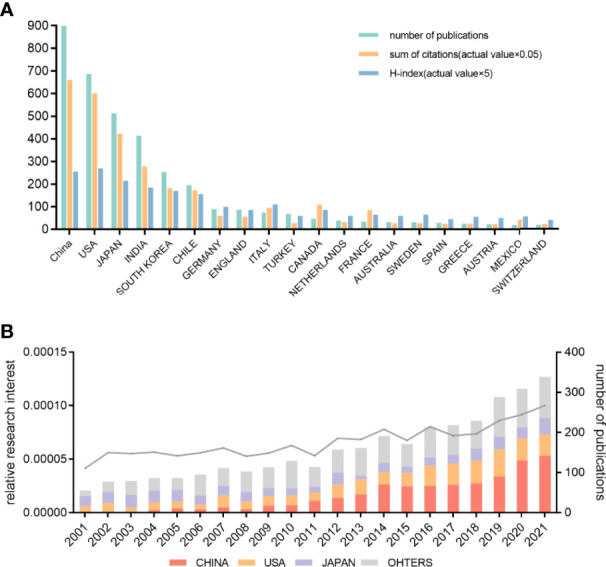
Contributions to the gallbladder cancer research of different countries or regions. **(A)** The number of publications, sum of citations (*0.05), and H-index (*5) of the top 20 countries or regions. **(B)** The number of publications worldwide and the top 3 countries per year are shown in the histogram. Line chart shows the time course of relative research interest.

**Figure 3:**
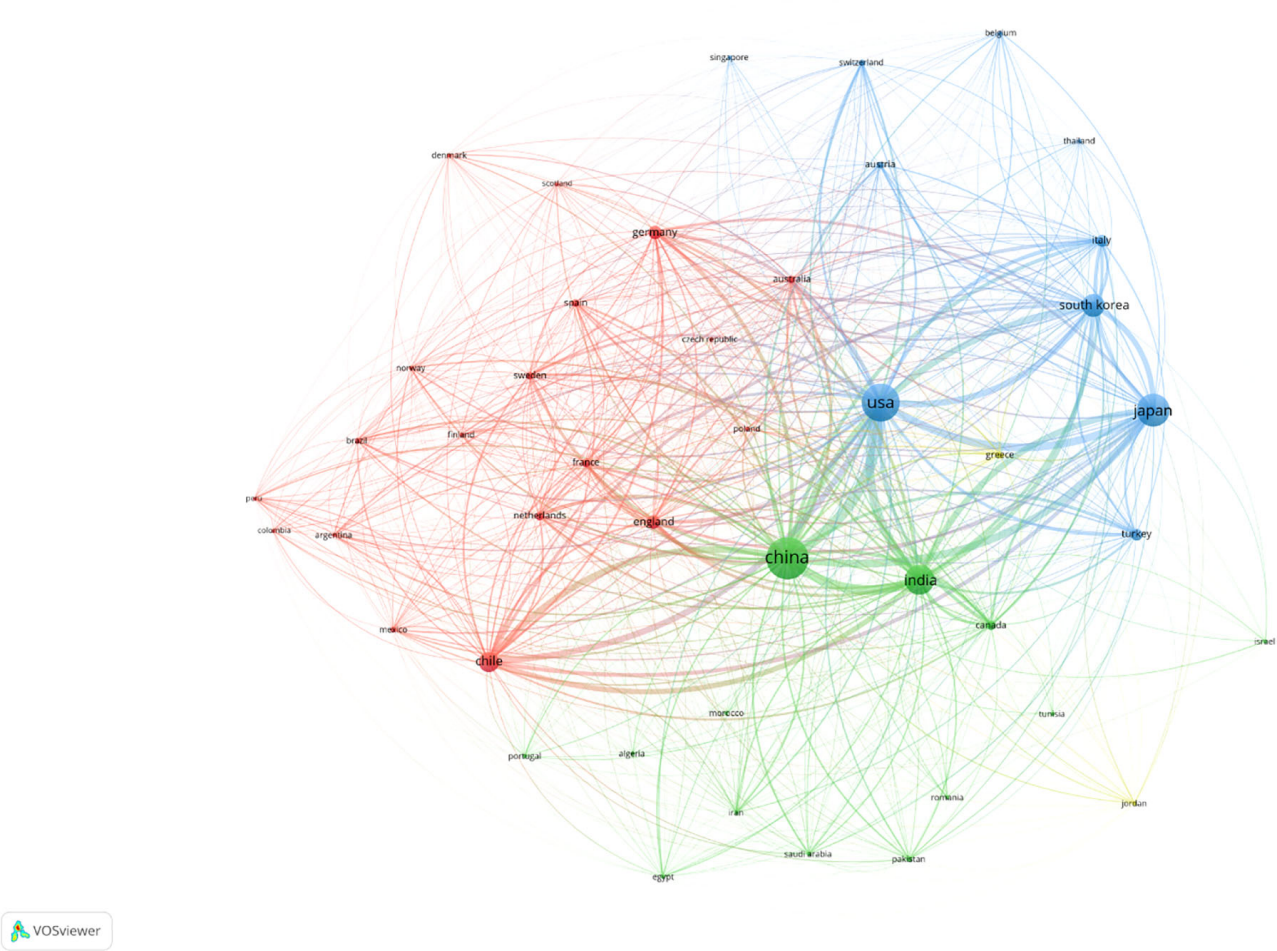
A visual network of countries publishing at least five gallbladder cancer research. All keywords were divided into four clusters and given different colors; the circle with a large sizer represented a higher frequency.

A total of 3,323 related publications from 2001 to 2021 were filtered and included in this bibliometric analysis (**Figure 1**). As shown in **Figure 2A**, China contributed the most publications (n = 900, 27.08%), followed by USA (n = 687, 20.67%), Japan (n = 513, 15.44%), India (n = 414, 12,46%), and South Korea (n = 253, 7.61%). Also, China and USA contributed publications with the highest number of citations in a minute difference (n = 13,206, 12,016, respectively). With regard to the number of publications per year, the most publications were issued in 2021 (n = 236, 7.10%). An increasing trend of publications per year is clearly demonstrated in **Figure 2B**. RRI represents the proportion of publications in GBC research compared to publications in all fields. The RRI rose from 0.0041% in 2001 to 0.0100% in 2021 (**Figure 2B**). There were three stages of RRI trend. In the first stage, from 2001 to 2011, the RRI shows a relatively stable trend, while in the second stage, from 2012 to 2018, the RRI increased to a new platform and kept stable. From 2019 to 2021, the third stage shows a rapidly increasing trend (**Figure 2B**). Moreover, excluding the countries that appear less than five times in all related publications, 44 countries meet the criterion and are analyzed by VOSviewer (**Figure 3**). The co-occurrence relations of the countries are classified into four clusters. Within these four clusters, there were three major clusters and their key countries: 1) China and India; 2) the USA, Japan, and South Korea; 3) Chile and European countries.

### Publication Growth Trend Predictions

**Figure 4:**
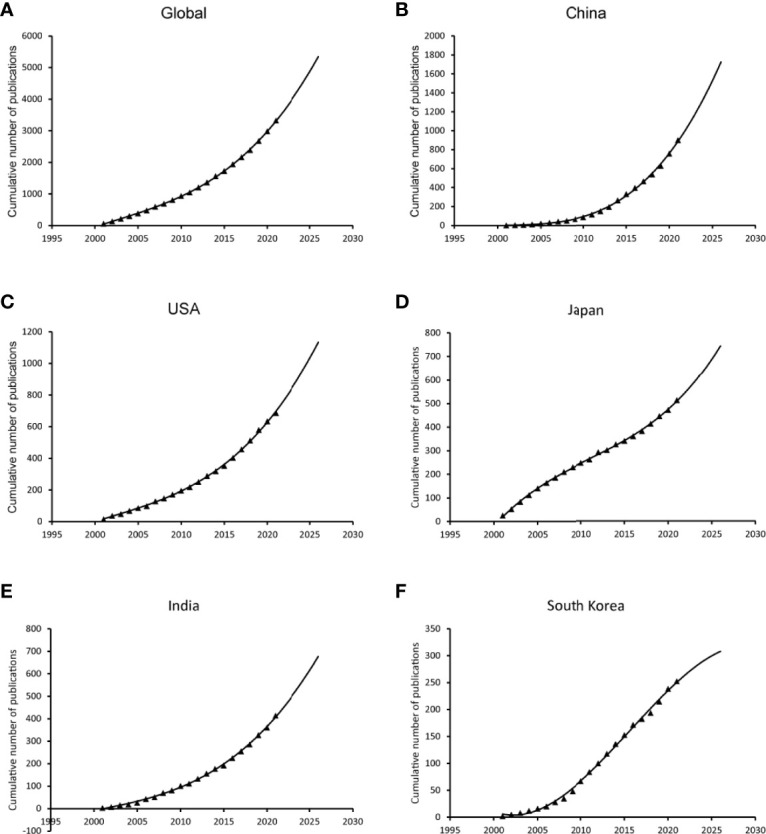
Fitting curves of publication growth trends.

The model fitting curves of the growth in GBC research illustrate that the number of publications grew significantly associated with time (**Figure 4**) and the trends of publications in the following 5 years were estimated according to the cumulative number of the articles published over the past 20 years. Apparently, the cumulative number of publications increases steadily around the world, including in USA and India. The cumulative number of publications in Japan grew slowly from 2001 to 2010 and increased rapidly during the latest decade, while the cumulative number of publications in South Korea shows a trend of slowing down after rapid growth in the previous decade. China has exhibited significantly faster growth, resulting in the contribution of the most publications.

### Journal Publishing Research and Institution Publishing Research

**Figure 5:**
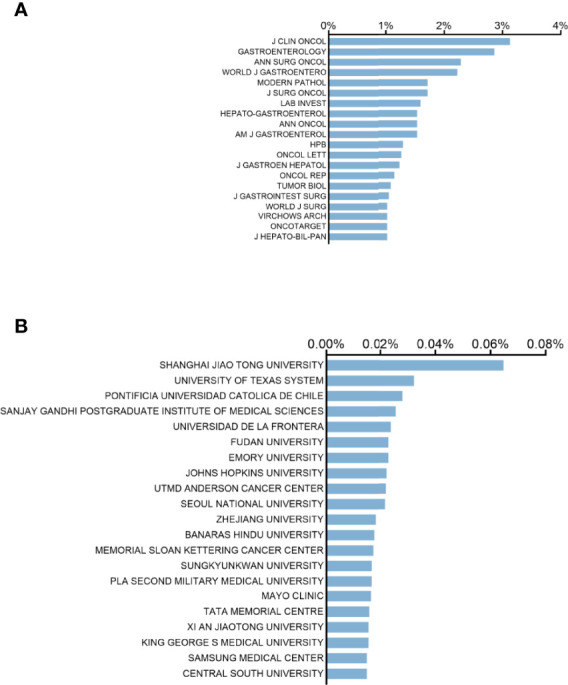
Distribution of journals and institutions focusing on gallbladder cancer. **(A)** Top 20 journals with the most publications in this field. **(B)** Top 20 institutions with the most publications in this field.

In terms of journals, the top 20 journals with the most publications in GBC research occupied one-third of the articles published in 2001–2021 (n = 1,040, 31.30%). Among them, Journal of Clinical Oncology published the most articles with 104 publications (**Figure 5A**). Gastroenterology comes in second with 95 publications, followed by Annals of Surgical Oncology, World Journal of Gastroenterology, and Modern Pathology with 76, 74, and 57 publications, respectively.

Shanghai Jiaotong University contributed the most publications (n = 215, 6.47%), far ahead of other institutions (**Figure 5B**). The University of Texas system ranked second with 107 articles published. Six of the top 20 institutions (Samsung Medical Center and Central South University are tied for 20th) are from China and USA, respectively, and four are from India. South Korean institutions and Chilean institutions contribute three and two, respectively.

### Authors’ Publication on GBC Research

As shown in **Table 1**, Roa JC of Pontificia Universidad Catolica De Chile (n = 89, 2.68%), Liu YB of Shanghai Jiaotong University (n = 87, 2.62%), and Quan ZW (n = 55, 1.66%) were the most productive authors in GBC research. Within them, Wu XS, of Shanghai Jiaotong University, has 2,045 citations with only 47 papers. The top 10 authors each have more than 1,000 citations. Notably, eight of the top 10 authors are from Shanghai Jiaotong University. Considering that cooperation between researchers can highly improve the efficacy and quality, the cooperation network between authors with more than 10 publications is presented in **Supplementary Figure 1**. As shown in **Supplementary Figure 1**, authors from Shanghai Jiaotong University have close cooperation.

**Table 1:** Top 10 authors with the most publications in gallbladder cancer research.

Authors	Country	Affiliation	Publications	Citations
Roa JC	Chile	Pontificia Universidad Catolica De Chile	89	1,343
Liu YB	China	Shanghai Jiaotong University	87	2,812
Quan ZW	China	Shanghai Jiaotong University	55	2,018
Mittal B	India	Sanjay Gandhi Postgraduate Institute of Medical Sciences	53	1,141
Li ML	China	Shanghai Jiaotong University	49	1,951
Wu XS	China	Shanghai Jiaotong University	47	2,045
Zhang YJ	China	Shanghai Jiaotong University	47	1,069
Hu YP	China	Shanghai Jiaotong University	46	1,792
Jiang L	China	Shanghai Jiaotong University	43	1,811
Gong W	China	Shanghai Jiaotong University	39	1,445

### Publications With High Academic Impact on GBC Research

Publications with high academic impact tend to be cited frequently. We used WOS to filter out the publications with the most citations and list them, as shown in **Table 2**. Several publications clearly illustrated that the management and epidemiology of GBC were on the list. Interestingly, research on the Keap1/Nrf2 signaling pathway and LncRNA CCAT1 identified the mechanism of chemotherapy resistance and carcinogenesis in GBC.

**Table 2:** Top 10 publications with the most citations in gallbladder cancer research.

Title	Corresponding authors	Journal	Publication year	Total citations
Epidemiology and molecular pathology of gallbladder cancer	Lazcano-Ponce, EC	CA-A Cancer Journal for Clinicians	2001	545
Gallbladder cancer: epidemiology and outcome	Shaffer, Eldon A.	Clinical Epidemiology	2014	511
Epidemiology of Gallbladder Disease: Cholelithiasis and Cancer	Shaffer, Eldon A.	Gut and Liver	2012	503
Gallbladder cancer worldwide: Geographical distribution and risk factors	Randi, G	International Journal of Cancer	2006	496
Carcinoma of the gallbladder	Misra, S	Lancet Oncology	2003	484
Is postoperative adjuvant chemotherapy useful for gallbladder carcinoma? A phase III multicenter prospective randomized controlled trial in patients with resected pancreaticobiliary carcinoma	Takada, T	Cancer	2002	436
Genetic alteration of Keap1 confers constitutive Nrf2 activation and resistance to chemotherapy in gallbladder cancer	Shibata, Tatsuhiro	Gastroenterology	2008	348
Patterns of initial disease recurrence after resection of gallbladder carcinoma and hilar cholangiocarcinoma - Implications for adjuvant therapeutic strategies	Jarnagin, WR	Cancer	2003	314
Gallbladder cancer: Lessons from a rare tumour	Gazdar, AF	Nature Reviews Cancer	2004	305
Long non-coding RNA CCAT1 promotes gallbladder cancer development *via* negative modulation of miRNA-218-5p	Quan, ZW	Cell Death & Disease	2015	288

### Analysis of Keywords on GBC Research

**Figure 6:**
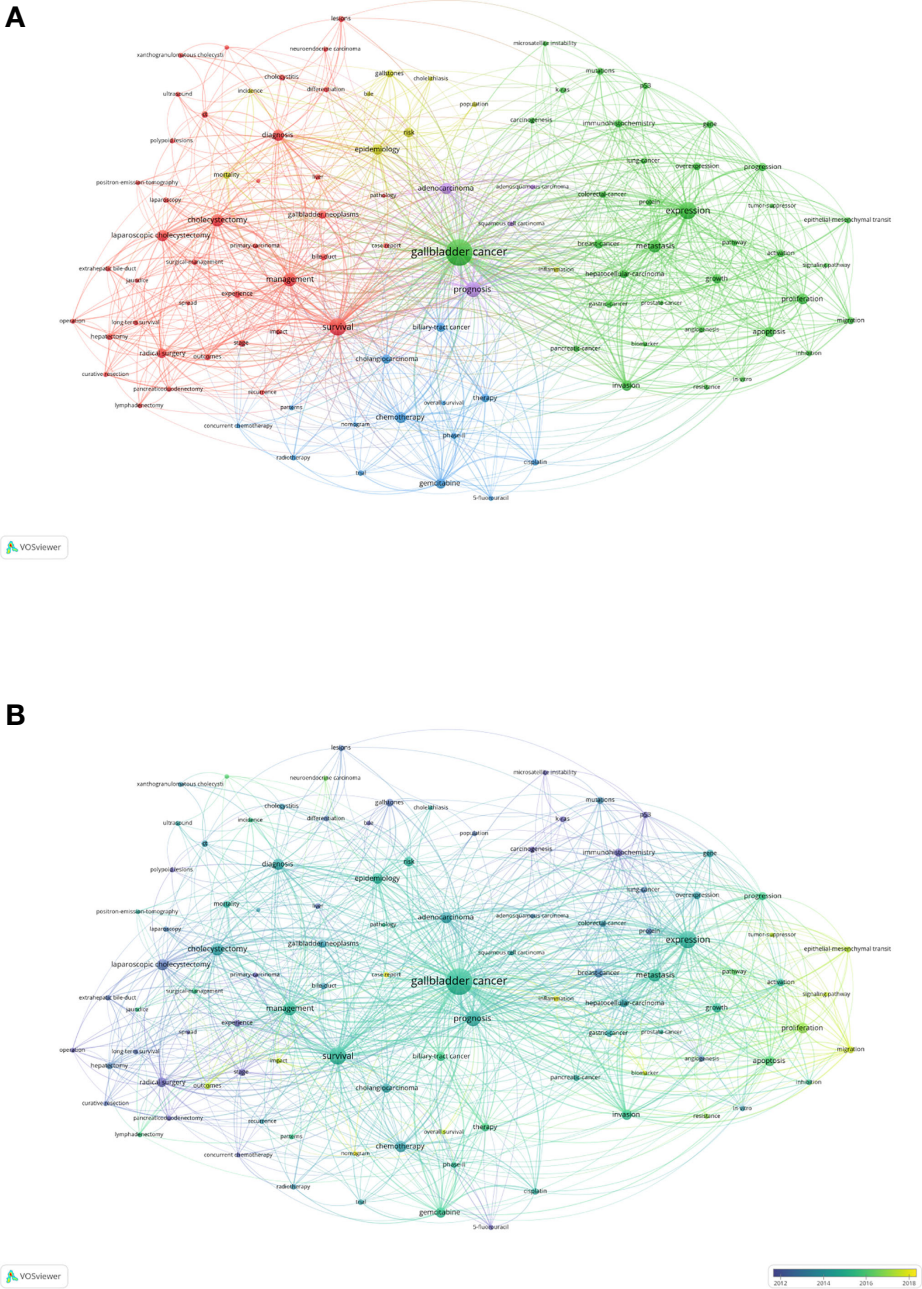
A visual network of the keywords in gallbladder cancer research. **(A)** Mapping of the keywords. All keywords were classified into five clusters and given different colors. The larger size of circle represented a higher frequency. **(B)** Distribution based on the average time of appearance. The color of circles represents the year of appearance. Yellow indicates recent appearance, and blue indicates early appearance.

All keywords that occurred more than 25 times were analyzed by using VOSviewer. After eliminating redundant synonyms and meaningless words, 101 keywords were identified and classified into five clusters: surgery therapy, mechanism research-related study, non-surgery therapy, epidemiology-related study, and pathology-related study (**Figure 6A**). Regarding surgery therapy, survival (n = 394), management (n = 222), cholecystectomy (n = 192), and diagnosis (n = 168) are the most frequent keywords. In mechanism research-related study, gallbladder cancer (n = 898), expression (n = 373), metastasis (n = 207), and proliferation (n = 143) are the most frequent keywords. With regard to pathology-related study, epidemiology-related study, and non-surgery therapy, prognosis (n = 290), adenocarcinoma (n = 163), chemotherapy (n = 156), gemcitabine (n = 141), epidemiology (n = 143), and risk (n = 120) are the most frequent keywords in their own clusters.

To illustrate the hotspots appearing in recent years on GBC research, we calculated the average appearing years (AAY) of keywords (**Figure 6B**). Keywords shown in yellow such as migration (cluster 2, AAY of 2017.4), biomarker (cluster 2, AAY of 2017.4), and nomogram (cluster3, AAY of 2019.6) appear most recently. Mechanism research-related study and non-surgery therapy may be a new research trend.

## Discussion

Our study analyzed 3,323 GBC publications published in the past two decades. Surprisingly, our research indicates that China contributed the most publications and citations and a high H-index value in the area of GBC research. This may be due to China being one of the countries with the highest burden of GBC (3). Following China, the USA also made great contributions to GBC research. From three to six, Japan, India, South Korea, and Chile contributed about one-third of total publications. These four countries are among the countries with the highest burden of GBC over the world, with Chile having a very high incidence of 5.6 cases per 100,000 people (3, 15, 16). Besides, the poor prognosis of GBC may be the reason for the large number of publications in GBC research. It is reported that the median survival time of GBC patients is less than 1 year and the 5-year overall survival is only 5% (17).

As illustrated in the network of countries’ cooperation, the highest link strength was between China and USA, which have extensive cooperation and exchanges in the new century. There are also frequent communications between China and India, China and Japan, and the USA and Japan, showing the international cooperation between these countries with numerous publications.

As shown in the cumulative curve of publications, global research on GBC has increased steadily over time. Notably, the time curve of China started off slowly but increased dramatically and showed the fastest growth rate. Nevertheless, the increase rate of South Korea’s time curve declined and increased mildly, which could account for the descendent trend of incidence in Korea from 2005 to 2015 (18, 19).

Journals in oncology and gastrointestinal diseases such as Journal of Clinical Oncology, Gastroenterology, and Annals of Surgical Oncology were the primary journals publishing research on GBC. World Journal of Gastroenterology also had a large number of publications. Therefore, it is reasonable to predict that future developments in the field will be more likely published in these journals. Among the 104 publications published by Journal of Clinical Oncology, 102 publications were consistent with abstracts, leading to change in clinical trials and therapy in GBC research (data not shown). Excluding meeting abstracts, World Journal of Gastroenterology published the most articles in GBC research (n = 74).

Within the top 20 institutions in surgical training, six institutions from China and USA, respectively, demonstrate their dominant status in this field. Regarding authors, Roa JC from Pontificia Universidad Catolica De Chile and Liu YB and Quan ZW from Shanghai Jiaotong University had published the most papers on GBC research. These scholars were considered to be pioneers in this field, and their studies will continue to influence future research development and guide cutting-edge research. Interestingly, as shown in **Table 1** and **Figure S1**, Liu YB’s team, Gong W’s team, and Wang J’s team contributed to most of the research published by Shanghai Jiaotong University. A cooperative but also competitive relationship between these three teams was clearly revealed by the network map.

A network analysis of literature keywords demonstrated the research interests and divided them into five clusters. The three major clusters were surgery therapy, mechanism research-related study, and non-surgery therapy, representing the clinical research and basic research. Radical surgery remains the only curative treatment for resectable GBC (20). Since only 10% of patients have the opportunity of a radical resection, systemic therapy became an important subject of research (21, 22). Along with the development and deeper perception of oncogenesis and perioperative management of GBC, a better paradigm of chemotherapies and more targeted therapy and immune therapy lead to a better prognosis for patients with GBC (9, 10, 23). Regarding the mechanism research-related study, the molecular mechanism in different hallmarks of GBC, especially the role and regulated pathway of high-frequency mutations of Erbb2, can bring us a deeper understanding and provide a novel therapeutic target for GBC (12, 24, 25). By time-series analysis, the network map showed that migration, biomarker, and nomogram had appeared most recently. This result demonstrated that mechanism study remains a hot research topic in GBC research and the mechanism in migration of GBC is a research priority, as lymph node metastasis is the key factor in the clinical staging of GBC. Additionally, because of the poor prognosis, finding better diagnostic and prognostic biomarkers and nomograms for prognosis and metastasis can improve the efficacy of personalized therapy and the survival of GBC patients. Bibliometrics is a quantitative analysis method based on mathematics and statistics. Using a visualization model can present the time sequence and clustering of keywords in the literature reflecting the interaction network, which is of great significance to scientific research evaluation. This study made an evidence-based visualization analysis of English publications to clarify the current hotspots of GBC research. However, the study still has limitations. The English publications are solely based on the WoSCC database with the exclusion of other English databases, which may cause some bias. Literatures with the non-standard use of keywords may lead to omissions in the literature retrieval based on these search strategies, which may affect the results and accuracy of cluster analysis. Moreover, the latest publications which do not have enough time to accumulate considerable citations were not included, which may partially affect our conclusions.

The study has demonstrated publication trends in GBC research. China and USA have contributed the most research and play important roles as the leading contributors. Other countries like Japan, India, South Korea, and Chile have also made great contribution to GBC research. Research in GBC has developed steadily and rapidly in the past two decades. By keywords analysis, the hotspots of GBC are mainly related to surgery therapy, mechanism research-related study, and non-surgery therapy. Elucidating deeper mechanisms in the migration of GBC cells, new biomarkers and highly effective nomograms will be the major problems and research directions in the future.

## Data Availability Statement

The original contributions presented in the study are included in the article/**Supplementary Material**. Further inquiries can be directed to the corresponding authors.

## Author Contributions

All authors made substantive intellectual contributions to the study. SS and LH design the conception of the study. SW, SS and LH modified the design of the study. GZ and ST collected the data. SW and WW performed the study and analyzed the data. SW and WW drafted the Introduction, Result and Discussion sections. GZ drafted the Methods sections. SW, SS and LH edited the manuscript. All authors have agreed to be accountable for all aspects of the work in ensuring that questions related to the accuracy or integrity of any part of the work are appropriately investigated and resolved. All authors contributed to the article and approved the submitted version.

## Funding

This research was funded by the National Natural Science Foundation of China (No. 82072682), Natural Science Foundation of Shanghai (21ZR1459100), and Science and Technology Commission of Shanghai Municipality (20DZ2254500).

## Conflict of Interest

The authors declare that the research was conducted in the absence of any commercial or financial relationships that could be construed as a potential conflict of interest.

## Publisher’s Note

All claims expressed in this article are solely those of the authors and do not necessarily represent those of their affiliated organizations, or those of the publisher, the editors and the reviewers. Any product that may be evaluated in this article, or claim that may be made by its manufacturer, is not guaranteed or endorsed by the publisher.

## Supplementary Material

Supplementary Figure 1 A visual network of the authors publishing more than 10 publications in gallbladder cancer research. All authors were divided into 9 clusters.
