# Supplementary figures and images for: Publication Trends of Research on Gallbladder Cancer During 2001–2021: A 20-Year Bibliometric Analysis
Source: Front Oncol. 2022 Jul 11;12:932797. doi: 10.3389/fonc.2022.932797 (PMC9309359; doi:10.3389/fonc.2022.932797)

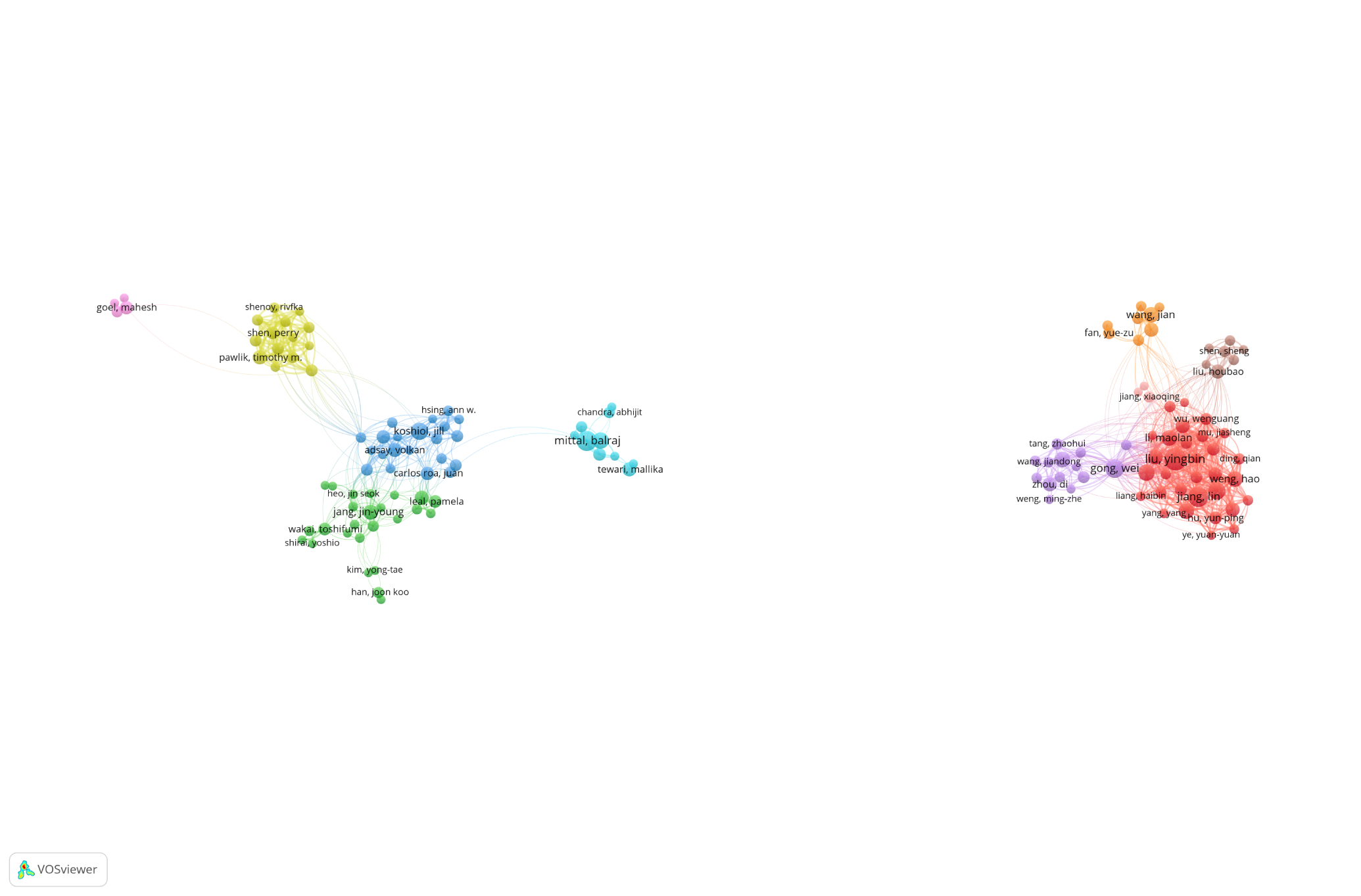

Supplement: Supplementary Figure 1 — A visual network of the authors publishing more than 10 publications in gallbladder cancer research. All authors were divided into 9 clusters. [file Image_1.tif]
